# The public health burden of obstructive sleep apnea

**DOI:** 10.5935/1984-0063.20200111

**Published:** 2021

**Authors:** Andre Faria, AJ Hirsch Allen, Nurit Fox, Najib Ayas, Ismail Laher

**Affiliations:** 1 Universidade Federal de Minas Gerais, Faculdade de Medicina - Pampulha - Belo Horizonte - Brazil.; 2 University of British Columbia, Department of Medicine, Faculty of Medicine - Vancouver - British - Columbia - Canada.; 3 University of British Columbia, Department of Anesthesiology, Pharmacology & Therapeutics, Faculty of Medicine - Vancouver - British - 'Columbia - Canada.

**Keywords:** Sleep Apnea, Motor Vehicle Accidents, Cognitive Dysfunction, Treatment, Health Burden, Economic Burden

## Abstract

Obstructive sleep apnea (OSA) is the most common respiratory disorder of sleep. The vast majority (>80%) of adults with moderate to severe OSA remain undiagnosed. The economic costs associated with OSA are substantial for both the individual and society as a whole; expenses are likely to be underestimated given that the disease remains undiagnosed in such a large percentage of individuals. The economic burden of motor vehicle collisions related to OSA alone is significant; it is estimated that 810,000 collisions and 1400 fatalities from car crashes in the United States were attributable to sleep apnea in 2000. The many health consequences of OSA include daytime sleepiness, reduced quality of life, decreased learning skills, and importantly, neurocognitive impairments that include impaired episodic memory, executive function, attention and visuospatial cognitive functions. Untreated OSA leads to numerous medical problems such as cardiovascular diseases that can potentially increase healthcare utilization. Untreated patients with sleep apnea consume a disproportionate amount of healthcare resources, expenditures that decrease after treatment. The gold-standard management of OSA remains treatment with CPAP (Continuous Positive Airway Pressure), which is effective in eliminating sleep fragmentation and preserving nocturnal oxygenation, thereby improving daytime sleepiness and quality of life. However, its impacts in reversing neurocognitive function are still uncertain. A significant impediment to CPAP effectiveness is low adherence rates (ranges from 50% to 75%). It is commonly accepted that CPAP improves excessive drowsiness; hence meliorates attention, and accumulating data suggest that CPAP improves a variety of other outcomes such as the risk of motor vehicle crashes.

## INTRODUCTION

**Figure 1:**
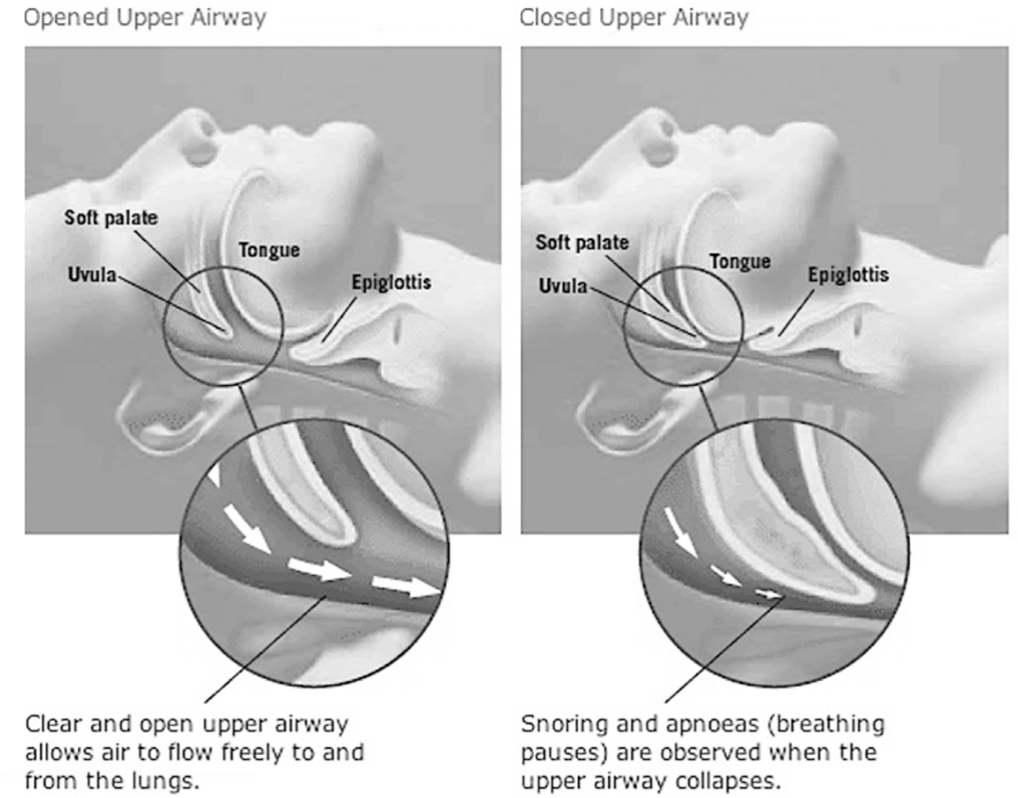
Airflow in a patent airflow compared to airflow in an obstruct airway.

Obstructive sleep apnea (OSA), the most common respiratory disorder of sleep^1^, refers to temporary cessations in breathing (apnea) or large reductions in breathing amplitude (hypopnea) caused by an obstructed or collapsed upper airway, both of which reduce blood oxygen (hypoxemia) and increase blood carbon dioxide (hypercapnia) levels^2,3^. The upper throat, tongue, and jaw muscles relax and interrupt normal breathing during OSA (Figure 1). A commonly used quantitative measure of OSA severity is the apnea/hypopnea index (AHI), which describes the total number of apnea/hypopnea episodes per hour (Table 1)^4^. An obstructed airway increases resistance to airway flow, resulting in a greater breathing effort and increased resistance to breathing pressure, leading to cessations (lasting 10 to 60 seconds) of breathing, causing arousals that are necessary for reopening of the airways^5^, and the resumption of breathing.

**Table 1.** Apnea/hypopnea index (AHI).

AHI score	Severity
<5	Normal
5-15	Mild
15-30	Moderate
>30	Severe

It is estimated that the vast majority (>80%) of adults with moderate to severe OSA remain undiagnosed^6^. At least 11-46% of obese women and 33-77% of obese men are diagnosed with OSA and its characteristic symptoms^7^. OSA is more common in males, in people affected by obesity and arterial hypertension. One telltale sign of OSA in many patients is habitual snoring. Other precipitating factors include low levels of thyroid hormone (free T4), smoking, and use of muscle relaxants such as benzodiazepines; however, the leading risk factor remains obesity^8,9^. The links between obesity and OSA are of particular concern given the global obesity epidemic^10^. Fat deposition around the upper airway narrows it and predisposes it to collapse. Furthermore, obesity reduces lung volumes, which also destabilize the upper airway by reducing the tethering effect of lung volume. However, it is important to note that reducing weight is not easily achievable in many patients, and OSA is not always resolved by weight loss alone.

Hampering research in the underlying mechanisms of OSA and the development of new therapeutic strategies is the lack of easy to use animal models that mimic key aspects of human OSA. The only animal to exhibit spontaneous OSA and snoring is likely the English bulldog, whose abnormal anatomy in the upper airways leads to hypopneas and fragmented sleep^11,12^. Other laboratory models of OSA include surgically producing airway occlusion and induction of intermittent hypoxia (by forcing rodents to breathe nitrogen for brief periods)^13,14^. However, the sequelae of OSA in most laboratory animals bear little resemblance to that in humans. The further discovery and strategic use of specific biomarkers are likely to allow a better prediction on the vascular and cognitive risks associated with OSA in the future. 

### The economic burden of OSA

The economic costs associated with OSA are substantial for both the individual and society as a whole; these economic costs are likely to be underestimated given that the disease remains undiagnosed in such a large percentage of individuals^15^.

Although it is difficult to quantify the exact economic burden of OSA, the number is in the range of billions of dollars per year in the United States alone. While the signs of OSA may not be as overt as in other chronic diseases, experts think its economic burden may be similar to that of diseases such as diabetes and asthma^15^. The economic burden of motor vehicle collisions related to OSA alone is significant, with the National Safety Council estimating that 810,000 collisions and 1,400 fatalities from car crashes in the United States were attributable to sleep apnea with an estimated cost of 15.9 billion dollars in 2000^16^. Researchers have devised a method of estimating the total cost of sleep disorders (mainly OSA, insomnia, and periodic limb movements) in various countries^17^. This method includes accounting for the direct cost of managing sleep disorders and their associated conditions, the indirect costs associated with work-related injuries, motor vehicle crashes and lost productivity, and the non-financial costs derived from the loss of quality of life and premature death (suffering). It is estimated that sleep disorders costs in the United States would be of a similar magnitude to that of diabetes, at or around 130 billion dollars per year^18^. 

### The public health burden of OSA

The public health impacts of OSA include work limitations and absenteeism, occupational injuries, motor vehicle injuries, cardiovascular disease, and reduced quality of life. Additionally, OSA is associated with neurocognitive impairments that include memory deficits, inability to concentrate, executive and motor dysfunction, and decreased alertness^19,20^. These OSA related impairments make it difficult for people to participate in the workforce and drive motor vehicles, and increase their use of the health care system^15,21^.

Neurocognitive deficits associated with OSA include impaired episodic memory, decreased attention, and executive and visuospatial cognitive dysfunctions^22,23^. Longitudinal studies in the general population have found OSA to be an independent risk factor for diagnose and the development of mild cognitive impairment (MCI) and dementia^24-31^. Additionally, studies in China found that patients with OSA had decrements in the visuospatial/executive function, attention, and delayed recall components of standardized neurocognitive exams. These decrements were associated with increasing OSA severity^32,33^. A publication by Sharma et al. (2018)^28^ found that OSA was associated with markers of increased amyloid burden over a 2-year follow-up period in a cohort of cognitively normal elderly men. The exact mechanisms by which OSA leads to cognitive impairment remain unclear. Two potential mechanisms include one where the neurocognitive effects are mediated by symptoms of OSA (e.g., disturbed sleep or sleepiness) and a second where the decrements are a consequence of direct neural damage mediated by nocturnal hypoxemia^34,35^. 

When it comes to the proposed pathophysiological mechanisms leading to neural damage and cognitive dysfunction, changes in nocturnal intracranial hemodynamics and oxygen saturation play a fundamental role. During an apneic episode, cerebral blood flow velocity increases progressively and then decreases sharply below baseline at the end of the episode^36^. Cerebral autoregulation protects the brain in healthy individuals by maintaining cerebral perfusion during wide excursions in blood pressure. However, this adaptive mechanism is impaired in patients with OSA, resulting in hypoperfusion in areas with a weak collateral circulation, such as the terminal arterial territories^36^. The impairments in cerebral autoregulation in patients with OSA can result in cerebral small vessel disease (C-SVD) and ischemic changes in white and gray matter^37,38^. The progressive worsening of C-SVD results in lacunar infarcts, white and gray matter lesions, and white matter fiber tract abnormalities^36,38^. Some regions of the brain, such as the prefrontal and frontal lobes, the basal ganglia, and the hippocampus, are more vulnerable to prolonged hypoxic-ischemic injuries^36^. Damage to these areas is associated with abnormal myelin and axonal integrity, which is related to mood disturbance and cognitive deficits. Prolonged hypoxic-ischemic damage to the frontal and prefrontal cortex is associated with executive dysfunction in patients with moderate to severe OSA^36^. 

On a molecular analysis, chronic intermittent hypoxia encountered by OSA patients increases reactive oxygen species (ROS) production and oxidative stress, which are associated to decreased synthesis and folding of neuroprotective and antiapoptotic factors^39^. Hypoxia plays an essential role in microscopic changes in the brain, as mostly shown in animal studies. A recent study suggests the endoplasmic reticulum stress response, mediated by ROS, as one of the potential causes of cognitive impairments in OSA^39^. Hypoxia stimulates the accumulation of unfolded proteins in the endoplasmic reticulum, promoting an inflammatory response and activation of apoptosis pathways in the brain. Moreover, there is lower expression of Bcl-2 and BDNF in neuronal tissue: these proteins are responsible for preventing apoptosis, and promoting neuronal survival and reposition, respectively. Intermittent hypoxia also diminishes oxidative phosphorylation, leading to poor maintenance of neuronal ion gradients^39^. When such changes occur in the hippocampus, there is a deficit in synaptic plasticity, cellular loss, and a reduction of neuronal excitability, which explain, somewhat, the memory dysfunctions^39,40^.

The neurocognitive impairments related to OSA leads to errors while driving and result in an increased risk of motor vehicle accidents^41^. A meta-analysis performed by Tregear et al., in 2011^42^, confirmed previous research indicating drivers with OSA have roughly twice the risk of crash as comparable drivers who do not. Additionally, they were able to identify some factors that may increase crash risk including BMI, increasing OSA severity (as measured by the AHI), and the severity of hypoxemia^42^.

The neurocognitive impairments discussed above have also been shown to lead to a high rate of collisions in patients who drive as a part of their occupations^41^. Howard et al. (2004)^41^ found that in a large sample (n=2,342) of commercial vehicle drivers, drivers with OSA (diagnosed based on symptoms) had an increased rate of self-reported MVC compared to controls (OR=1.30)^41^. This increased risk extends to occupational injuries (OI). The vast majority of studies investigating the relationship between OSA and OI have shown a significant relationship^43-45^. These studies have indicated OI rates are between 2 and 3 times higher in OSA patients^45^.

Furthermore, two longitudinal studies identified OSA as an independent risk factor for depression, in which the odds for developing depression were increased 2.0-fold (95% confidence interval [CI]: 1.4-2.9) in participants with mild OSA and 2.6-fold (95% CI: 1.7-3.9) in those with moderate to severe OSA^46,47^. Ischemic alterations in cerebral microvascular structures linked to depression are related to the “hypoperfusion” mechanism of vascular depression^36^. This hypothesis implies that impairment in hemodynamics and cerebral autoregulation leads to cerebral perfusion deficits, altered regional brain function, and white matter hyperintensities. Also, the “disruption” mechanism of vascular depression suggests that the harm to particular fiber tracts and neural circuits, especially the frontostriatal and limbic systems, results in hindered neural connections that regulate mood and cognition. Specifically, aggravated damage to the superior longitudinal and uncinate fasciculi is associated with more severe depression and executive dysfunction^36^. 

Additionally, observational studies show that workers with OSA have increased work absences as well as decreased performance while at work compared to healthy individuals^48-50^. This is not surprising given that OSA is associated with deficits in verbal and executive function, and problem-solving. Excessive daytime sleepiness is strongly associated with work impairment in sleep apnea populations. The impairment in work productivity due to excessive daytime drowsiness is similar to that reported for other chronic conditions such as diabetes and arthritis^50-52^.

Untreated OSA also leads to numerous medical concerns such as cardiovascular disease that can potentially increase healthcare utilization. Many studies suggest that OSA is as an independent risk factor for the development of cardiovascular diseases (Table 2). Additionally, a cohort study suggests OSA as an independent risk factor for reduced endothelium regeneration and augmented apoptosis of endothelial cells. These early endothelial alterations may not only underlie accelerated atherosclerosis and increased cardiovascular risk in OSA, but also predispose to vascular dementia and neurocognitive impairment^53^. Conditions such as hypertension, type 2 diabetes, atrial fibrillation, atherosclerosis, stroke, and heart failure are not only harmful due to their potential to lethality, but are predisposition factors for other diseases. It is generally accepted that untreated patients with sleep apnea consume a disproportionate amount of healthcare resources and that healthcare expenditures decrease after treatment. In the US it is estimated that a person with OSA costs the healthcare system about twice as much annually as a person without OSA ($2,720 vs. $1,384)^54^.

**Table 2.** Cardiovascular events.

Cardiovascular Events	
Hypertension	++ HR = 1.8 by 3.0 ~foldm55
Diabetes/Insulin Resistance	++ OR = 1.4656
Atrial Fibrillation	+ OR = 4.0257
Heart Failure	+ OR = 2.3858
Atherosclerosis	++ OR = 3.2159
Stroke	++ HR = 1.9460

Notes: Quality of evidence:

++ Consistent data from one large study or several small studies with results adjusted for confounding factors;

+ Consistent data from one large study or several small studies.

### Current OSA treatment strategies

**Weight loss and lifestyle interventions:** a recent systematic review estimated that nearly 38% of the world population suffers from OSA and that 60% of moderate to severe cases are attributable to obesity^61,62^. Related to this are the most recent practice guidelines from the American Academy of Sleep Medicine that strongly suggest alternative or combined behavioral interventions, including weight loss (through dietary approaches and exercise), sleep hygiene, and avoidance of alcohol and tobacco consumption^63,64^.

Several systematic reviews and meta-analysis summarized the findings of studies on the effects of weight loss interventions on OSA^65-70^. The main conclusion of these studies is that weight loss reduced primary outcomes such as AHI, oxygen desaturation index, and excessive day sleepiness. However, the small number of number of studies limited the standardization of the findings. Furthermore, the potential effects of other lifestyle components, and additional OSA parameters (oxygen saturation mean, arousal index, sleep continuity, and neurocognitive domains), were not considered and broader investigations are still needed.

Recent evidence from a meta-analysis revealed that lifestyle interventions caused significant reductions of all primary OSA outcomes (AHI, ODI, and EDS) and exhibited medium to high improvements in arousal index, oxygen saturation mean, and sleep efficiency^71^. Even there was not a full remission of OSA, the reductions of these parameters are clinically relevant. The improvements in hypoxia and reductions of daytime drowsiness by lifestyle interventions could not only reduce cognitive impairments but also diminish occupational and risks of motor vehicle accidents. Although this study indicates the potential beneficial effects of lifestyle intervention on OSA, future well-designed RCTs with longer follow-up periods are required to further support this evidence. Moreover, a wider analysis of lifestyle interventions on the clinical consequences of OSA, such as neurocognitive deficits and cardiovascular diseases, are needed to confirm therapeutic benefits.

There is insufficient evidence to support weight loss and lifestyle interventions as independent OSA therapies. Furthermore, mitigating the effects of obesity, alcohol consumption, and smoking for OSA does not treat sleep apnea itself, but rather may act to only attenuate aggravating factors. Nonetheless, current evidence regarding the beneficial effects of weight-loss suggests lifestyle modifications as a promising strategy when used in combination with other therapeutic options.

**Continuous positive airway pressure (CPAP):** nasal continuous positive airway pressure (CPAP, developed in 1981) therapy is considered the first-line treatment for moderate to severe OSA^72^. CPAP prevents the upper airway from collapsing by establishing a positive pressure in the pharynx (consisting of the nose, upper and lower airway passages) during sleep. CPAP eliminates sleep fragmentation and preserves nocturnal oxygenation, thereby improving daytime sleepiness, quality of life, and neurocognitive function^73^. One of the most significant impediments to CPAP effectiveness is poor adherence (ranges from 50% to 75%), often citing discomfort with the apparatus as the reason for noncompliance^74^. Regular use of CPAP increases vigilance, since it reduces daytime drowsiness, and accumulating data suggest that CPAP improves a variety of other outcomes such as motor vehicle crashes^75^.

As was discussed above, patients with mild, moderate, and severe OSA syndrome have been shown to have an increased rate of traffic accidents and higher rates of personal injury associated with those accidents^75^. Worse performances in driving simulators among subjects with untreated OSA were also identified^76-78^. Several studies have shown improvement in driving performance after treatment with CPAP^76,77,79^. It is likely that the CPAP therapy reduces daytime sleepiness, therefore improving driving performance and reducing the risk of accidents. These treatment benefits are highly likely to be highly dependent on treatment adherence^80^.

The benefits of CPAP therapy on neurocognitive impairments are still unclear (Table 3). There is agreement that CPAP reduces daytime sleepiness^(22,76,77,79-87.)^ Several studies indicated that neurobehavioral deficits (except vigilance, work memory, and executive functioning) could be also partially or fully be reversed by OSA treatment^81-83^. A randomized control trial with 141 patients concluded that CPAP intervention restored verbal fluency, psychomotor performance, complex cognition, and general memory function of mild OSA individuals to the control levels^83^. A randomized crossover trial also suggested an enhancement in speed deviation and in reaction times to divided attention tasks during a driving simulation^84^. A randomized controlled trial (with 32 patients) indicated significant improvements in gray matter volume in the hippocampus soon (as short as three months) after treatment with CPAP^85^.

**Table 3.** Summary of studies examining the effects of CPAP therapy on neurocognitive functions.

Author (year) [level of evidence]	Driving	Attention	Vigilance	Work memory	Executive function	Psychomotor function	Cognitive function	Depression/mood	Verbal fluency	Daytime sleepiness
Findley et al. (2000) ^75^ [3b]	+	NA	NA	NA	NA	NA	NA	NA	NA	+
Turkington et al. (2004) ^76^ [2b]	+	NA	NA	NA	NA	NA	NA	NA	NA	+
Antonopoulos et al. (2011) ^78^ [1a]	+	NA	NA	NA	NA	NA	NA	NA	NA	+
Vakulin et al. (2011) ^79^ [2b]	+ * *	NA	NA	NA	NA	NA	NA	NA	NA	NA
Engleman et al. (1998) ^80^ [1b]	+	NA	NA	NA	NA	NA	NA	NA	NA	+
Henke et al. (2001) ^81^ [1b]	+ *	NA	NA	+ *	-	NA	+ *	NA	NA	+ *
Jenkinson et al. (1999) ^82^ [1b]	NA	NA	NA	NA	NA	NA	+	NA	NA	+
Jackson et al. (2018) ^83^ [1b]	NA	NA	NA	-	-	+	+	+	+	+
Phillips et al. (2013) ^84^ [1b]	+	+	+	+	NA	+	+	-	+	+
Canessa et al. (2011) ^85^ [1b]	NA	NA	+	+	-	+	-	+	+	+
Kylstra et al. (2013) ^86^ [1a]	NA	+	-	-	-	NA	-	+	-	+
Kushida et al. (2012) ^87^ [1b]	NA	-	-	-	+	-	-	NA	NA	+

Notes: Symbols to indicate the sort of evidence:

+ Evidence of betterment;

- No evidence of betterment

NA - No assessment of the cognitive domain;

* Results statistically significant comparing treated OSA patients with no treated patients. But the improvements were not significant when compared to the sham-CPAP group.

The residual changes in vigilance and work memory reported in different studies could be related to comorbidities such as depression, temporal differences in the neurocognitive repair processes, or structural changes in brain regions responsible for those tasks. Symptoms and the remaining deficits not fully corrected by CPAP therapy were not related to the frequency and length of CPAP use, making it difficult to implicate different repairing times^83,84^. For example, some patients may have experienced a placebo effect, as reflected in an apparent development in self-reported OSA symptom severity and some of the neuropsychological tasks, as confirmed by the lack of difference from the sham-CPAP intervention^81^. They may also have tried harder or felt more competent in completing the neuropsychological tests. Another possibility is that the tasks used were not sensitive enough to detect the small differences that may have existed between the different CPAP usage times. Further, although there seemed to be an enhancement in gray matter volume, it was an isolated finding from a small study^85^, and additional evidence from larger randomized controlled trials are needed.

Although the prevalence of depression seemed to improve after treatment, its impact on neurobehavioral functioning is difficult to determine as the main etiologic factors for the impairments are unkown^83,84,86,87^. OSA and depression are both independently related to obesity, increasing age, and adverse lifestyles^88^. This means that both conditions share common symptomatology such as daytime sleepiness, insomnia, and fatigue. Consequently, improvements of AHI in after CPAP use may not necessarily result in a decrease of depressive symptoms, since other factors (e.g., obesity and unhealthy habits) remain. In turn, the anxiety and depression scales frequently used to measure baseline and post-treatment mood disturbances (e.g., Hamilton depression rating scale, Beck depression inventory, and Beck anxiety inventory) usually include somatic symptoms also caused by OSA - such as cardiovascular palpitations, headaches, gastrointestinal and genitourinary disturbances, loss of energy and libido, and fatigue. Therefore, high scores of these symptoms at baseline may not necessarily signify the presence of depression in patients with OSA. Further efforts to include anxiety and depression measures through valid scales adjusted to patients - focusing on non-overlapping symptoms such as cognitive-affective symptomatology - are needed to establish the presence of anxiety and depression disturbances in patients with OSA and also to clarify the effectiveness of CPAP therapy in reducing these specific and adverse outcomes and associations with neurobehavioral deficits.

A meta-analysis including 13 studies (554 patients with OSA) did not find evidence for any neurocognitive improvement after CPAP usage besides augmented attention^86^. The apnea positive pressure long-term efficacy study (APPLES), a 6-month, randomized, double-blind, 2-arm, sham-controlled, and multicenter trial, failed to show significant amelioration in neurocognitive restoration for attention, psychomotor function, memory, and learning, although there was a slight improvement in executive functioning, after two months of CPAP use^87^. 

**Oral appliances:** oral appliances (OA) to treat OSA fall into two broad categories: mandibular advancement splints (MAS), also known as mandibular repositioning devices, and tongue repositioning or retaining devices. MAS devices aims to advance the mandible slightly forward and so enlarge the upper airway and prevent it from collapsing^89^. Similarly, tongue-repositioning devices suction the tongue forward to prevent it from falling back and obstructing the airway during sleep. Selecting the most appropriate oral appliance, fitting, and determining the optimal degree of lower jaw advancement, which varies considerably between patients, is a complex process that requires special training in oral appliance therapy^90,91^.

The literature concerning the effects of oral appliances on treating neurobehavioral deficits in OSA patients is scarce. A randomized three-way crossover trial showed that both CPAP and MAS were superior to placebo in improving executive cognitive function and had no additional therapeutic effects on neurobehavioral domains^92^. The results were obtained by the paced auditory serial addition test (PASAT) that assesses information-processing speed and working memory, functions directly hampered by sleep fragmentation, and daytime drowsiness. Moreover, a randomized controlled crossover trial reported that while there were no improvements in neuropsychological domains (attention/working memory, verbal memory, visuospatial or executive functioning), treatment with the mandibular advancement splint improved performance on a test of vigilance/psychomotor speed^93^. The findings in both studies have not improved proportionally to the CPAP usage time and were associated with the betterment of daytime sleepiness.

Multiple randomized trials compared the treatment with oral appliances to CPAP, and reported improvements of the AHI and oxygen saturation with CPAP^94,95^. Regarding hypoxemia, allegedly the main etiologic factor for the neurocognitive impairments, CPAP has greater therapeutic potential than oral appliances. However, use of oral appliances has better compliance^94,95^. Further, the Federal Motor Carrier Safety Administration of the United States does not recommend the use of the OA in the treatment of OSAS for professional drivers as there is little evidence that for a reduction of accidents with OA^96^. 

**Surgery:** the most common surgical procedure for OSA is uvulopalatopharyngoplasty (UPPP), which removes excess tissue at the back of the throat (tonsils, uvula, and part of the soft palate)^97^. Various complications can occur with the procedure, and the success rate on average is 40 percent (success defined as the ratio postoperative AHI/preoperative AHI≤0,50 and post-operative AHI≤10 apneas per hour)^98^. The long-term side effects and benefits of UPPP are not known and there may be difficulties tolerating CPAP if it is needed after the procedure. In children, common procedures include the removal of adenoids and tonsils and correction of structural deformities. Younger patients may benefit from these surgical procedures more than older patients^99^. The benefits of surgical procedures on reducing AHI and daytime sleepiness, and reducing cognitive impairment, are unkow^100-102^. 

**Genioglossus stimulation:** this recently devised treatment focuses on the decreased neural drive to the upper airway muscles during sleep that causes airway closure in obstructive apnea. The genioglossus muscle is the largest upper airway dilator muscle. Electrical stimulation of the hypoglossal nerve (which innervates the genioglossus muscle) improves upper airway patency, which causes protrusion of the tongue^103^. Several companies have developed hypoglossal nerve stimulation (HGNS) devices. The Inspire HGNS device (Inspire Medical Systems, Maple Grove, MN, USA) has the most supporting data. This device has a neurostimulator that is implanted under the skin in the upper chest (similar to a cardiac pacemaker), with a stimulation electrode placed on the hypoglossal nerve and a sensing lead placed between the internal and external intercostal muscles to detect ventilatory effort^103^. This fully implantable system senses breathing patterns and delivers stimulation to maintain airway patency during sleep. The device is activated before bedtime. One shortcoming of this method is its requirement of an implanted battery, the replacement of which requires surgery^104^. Multiple studies report that the Inspire Medical Systems device lowers AHI averages and excessive daytimedrowsiness^105-107^. Further evidence regarding its benefits over neurobehavioral deficits is needed.

## CONCLUSIONS

Our understanding of OSA and its association with the economic expenditures on public health continues to evolve. Our survey of the literature indicates that OSA increases car accidents rates, cardiovascular risks, and induces neurocognitive impairments. However, it is difficult to establish a causal effect of OSA on neurobehavioral deficits, as the pathophysiological pathways are uncertain. Future studies should focus on risk stratification of OSA patients related to adverse neurocognitive outcomes. Studies to identify new OSA-specific biomarkers of cognitive deficits are still in their infancy.

There is a need for large-scale, randomized, well-controlled studies on OSA therapies (CPAP) on restoring neurobehavioral domains such as executive functioning, work memory, vigilance, verbal fluency, cognitive function, and psychomotor performance. The impact of the various therapeutic approaches on depression, and its repercussions on neurocognitive deficits, also needs further analysis.

In addition, to finding innovate ways to improve compliance with CPAP, there are also opportunities in the use of machine learning in this area. For instance, our single-center study indicates improved adherence with CPAP with a web-based telemedicine monitoring system^108^. 
